# Short and Long-Term Effectiveness of Ustekinumab in Patients with Crohn’s Disease: Real-World Data from a German IBD Cohort

**DOI:** 10.3390/jcm8122140

**Published:** 2019-12-04

**Authors:** Alica Kubesch, Laurenz Rueter, Karima Farrag, Thomas Krause, Klaus Stienecker, Johannes Hausmann, Natalie Filmann, Axel Dignass, Jürgen Stein, Irina Blumenstein

**Affiliations:** 1 Department of Medicine 1, Goethe University Hospital, 60590 Frankfurt a.M., Germany; laurenz.rueter@web.de (L.R.); johannes.hausmann@kgu.de (J.H.); irina.blumenstein@kgu.de (I.B.); 2 Interdisciplinary Crohn-Colitis Centre Rhein-Main, 60590 Frankfurt a.M., Germany; farrag@gmx.de (K.F.); j.stein@em.uni-frankfurt.de (J.S.); 3 Gastroenterological Practice, 34117 Kassel, Germany; krause@go-ks.de; 4 Gastroenterological Practice, 36039 Fulda, Germany; k.stienecker@gmx.de; 5 Institute of Biostatistics and Mathematical Modeling, University Hospital, Goethe University, 60590 Frankfurt a. M., Germany; filmann@med.uni-frankfurt.de; 6 Department of Internal Medicine 1, Agaplesion Markus Krankenhaus, 60590 Frankfurt a. M., Germany; Axel.Dignass@fdk.info

**Keywords:** IBD, Crohn’s disease, biologics, ustekinumab, real-world data, efficacy

## Abstract

Background and Aims: The IL-12/23 inhibitor ustekinumab (UST) opened up new treatment options for patients with Crohn’s disease (CD). Due to the recent approval, real-world German data on long-term efficacy and safety are lacking. This study aimed to assess the clinical course of CD patients under UST therapy and to identify potential predictive markers. Methods: Patients with CD receiving UST treatment in three hospitals and two outpatient centers were included and retrospectively analyzed. Rates for short- and long-term remission and response were analyzed with the help of clinical (Harvey–Bradshaw Index (HBI)) and biochemical (C-reactive protein (CRP), Fecal calprotectin (fCal)) parameters for disease activity. Results: Data from 180 patients were evaluated. One-hundred-and-six patients had a follow-up of at least eight weeks and were included. 96.2% of the patients were pre-exposed to anti- TNFα agents and 34.4% to both anti-TNFα and anti-integrin antibodies. The median follow-up was 49.1 weeks (95% CI 42.03-56.25). At week 8, 51 patients (54.8%) showed response to UST, and 24 (24.7%) were in remission. At week 48, 48 (51.6%) responded to UST, and 25 patients (26.9%) were in remission. Steroid-free response and remission at week eight was achieved by 30.1% and 19.3% of patients, respectively. At week 48, 37.6% showed steroid-free response to UST, and 20.4% of the initial patient population was in steroid-free remission. Conclusion: Our study confirms short- and long-term UST effectiveness and tolerability in a cohort of multi-treatment-exposed patients.

## 1. Introduction

Over the past decade, significant advances have been made in the medical treatment of patients with CD. The introduction of anti-tumor necrosis factor (TNF) and anti-integrin antibodies represent milestones in the recent treatment history [1,2]. Although being potent medications for treating CD, primary and secondary loss of response, as well as adverse events such as infections or allergic reactions are common, leading to a discontinuation of the therapy [3,4]. In light of the growing insight into the inflammatory signaling pathway involved in inflammatory bowel disease (IBD), newer therapeutic agents aim to act more specifically and to achieve a more targeted inflammation control. There is a growing demand for novel therapeutic agents targeting alternative disease mechanisms [5].

Ustekinumab (UST) is a monoclonal antibody targeting the p40 subunit of IL-12 and IL-23 and is a new treatment option for CD [6]. It has been approved for the treatment of CD in the European Union and the US since September 2016.

Several studies have provided real-world data on the safety and efficacy of UST. Due to its rather recent approval, most studies evaluate short-term effectiveness [7] only, are retrospective, or do not include the intravenous induction regimen [8,9]. Only a few studies provide insight into long-term effectiveness [10,11] or are prospectively conducted [12]. However, comparing this data is challenging, as the definition of remission and response is not consistent throughout the publications. Often only the clinical response or remission rates are considered.

Therefore, our main objective was to evaluate the real-world efficacy of long-term treatment with UST after the recent approval of the drug in Germany with the help of a combination of parameters for clinical and biochemical disease activity.

## 2. Methods

### 2.1. Study Design

Five centers, three hospital-based IBD, and two outpatient centers participated in this study. The patients were followed up at the treating physician’s discretion, at least every eight weeks for their subsequent dose of UST. Clinical disease activity indices and laboratory parameters relevant for disease monitoring were recorded at baseline and after 8, 16, 32, 48, 56, 72, and 88 weeks or until the medication was discontinued. Patient-specific data was obtained with the help of the electronic or paper-based patient file. The data was analyzed retrospectively.

### 2.2. Study Population

In this multicenter study, all patients with CD, receiving treatment with UST, and a minimum follow-up of at least eight weeks from their initial UST infusion were included in our study. All patients had to be diagnosed with CD according to standard criteria and had to be ≥18 years old. There were no other exclusion criteria. The initial intravenous (IV) UST dose was weight-adjusted (260 mg < 55 kg, 390 mg 55 kg–85 kg, 520 mg > 85 kg), and the first subcutaneous (SC) dose was 90 mg and given eight weeks after the first UST dose. Further UST injections were given either at an 8 or 12 weeks interval at the treating physician’s discretion. Patients with overall disease activity (i.e., either Harvey Bradshaw Index (HBI) > four and/or fecal calprotectin (fCal) > 250 with/without elevated C-reactive protein (CRP) were included in the evaluation of the efficacy.

### 2.3. Study Variables

Baseline characteristics included age, gender, disease duration, behavior, and location (Vienna Classification) [13]; medication previous and concomitant; surgery; complications; and disease severity. The treatment response was assessed with the help of the HBI [14,15] (HBI ≤ 4 remission, 5–7 mild activity, 8–16 moderate activity, >16 severe activity) as well as biochemical parameters of disease activity such as fCal and CRP. We considered this to be a combined remission, as both clinical and biochemical parameters for disease activity were taken into consideration. We aimed to record clinical and laboratory parameters for disease activity at every time point. Possible complications or adverse events were also recorded.

### 2.4. Definitions and Outcomes

The primary objective of this study was to determine the number of patients in combined remission at week 48. Secondary outcomes were: remission at weeks 16, 32, 48, 56, 72, and 88 as well as clinical response at weeks 8, 16, 32, 48, 56, 72, and 88. Achieving remission was compared between anti-integrin exposed and naïve patients as well as between patients with or without concomitant immunosuppressive medication at baseline. Furthermore, we aimed to determine predictors for remission at week 8 and 48. Remission was defined as an HBI ≤ 4 and/or fCal < 250 µg/g (if available). When there was a discrepancy between HBI and fCal, remission was not assumed in these cases. Clinical response was defined as a significant HBI reduction by at least three points or a fCal reduction of at least 50% or < 250 µg/g. The time of follow-up was based on the date of the initial intravenous UST application until the last documented visit. Patients that either stopped UST treatment due to adverse events, primary or secondary non-response, and having no long-term sustained remission were considered as treatment failures and non-responders; furthermore, if relevant information was missing for a certain time point, the patient was not included in the evaluation of this visit. The rates for response and remission were calculated using the initial baseline population with disease activity (i.e., *n* = 93) throughout the study for better comparability.

### 2.5. Ethics Statement

Approval for this retrospective study was obtained from the local Ethics Committee of the University Hospital Frankfurt (file number 58/19).

### 2.6. Statistical Analyses

Statistical analyses were conducted using IBM SPSS Statistics Version 22.0 (International Business Machine Corporation, Endicott, NY, USA). *p*-values ≤ 0.05 were considered to be statistically significant. All tests are two-sided. Associations of outcomes with dichotomic variables were assessed in binary logistic regression models. After univariate analyses, multivariate analyses were performed for significant associations. Multivariate models were obtained by backward selection, using a *p*-value > 0.1 for removal from the model. To determine UST drug continuation over the study time period, the Kaplan–Meier method was applied. Analyses were performed with intention-to-treat (i.e., patients being on UST). If a patient discontinued UST or was lost to follow-up, it was considered as a treatment failure for further analysis.

## 3. Results

### 3.1. Baseline Characteristics

A total of 106 patients were included in this retrospective cohort study. Ninety-three patients (87.7%) had active disease, whereas 13 patients (12.3%) were in remission at baseline, and thus not included in the efficacy analysis. Sixty-two patients (58.5%) were female, and the median disease duration was eleven years (range 2–39). Thirty-five patients (38%) had ileocolonic disease; a penetrating phenotype was documented in 43 patients (44.8%). One hundred and two patients (96.2%) had at least one previous biological treatment. Forty-six patients (43.4%) had more than one previous anti-TNFα treatment. Thirty-six patients (34.4%) were previously exposed to integrin inhibitors and 36 patients (34.4%), to both anti-TNFα and anti-integrin antibodies. Ninety-five patients (89.6%) were immunosuppressant (i.e., Azathioprine, 6-Mercaptopurine, and Methotrexate) experienced, and last, a total of 38 patients (35.8%) started UST with concomitant corticosteroid therapy. The baseline characteristics of the included patients are shown in Table 1.

### 3.2. Disease Efficacy Follow-up

Ninety-three patients (88.7%) had active disease at baseline. The median HBI was 8 (range: 0–26), CRP 0.85 mg/dl (range: 0.1–12.2) an fCal µg/g 539 (range: 5–2100). Of the total amount of patients, 78 were followed up for at least eight weeks and had sufficient information concerning disease activity and were thus included in the efficacy analysis. The median follow-up in this study was 49.1 weeks (95% CI 42.03–56.25).

### 3.3. Combined Response and Remission Analysis

**Figure 1:**
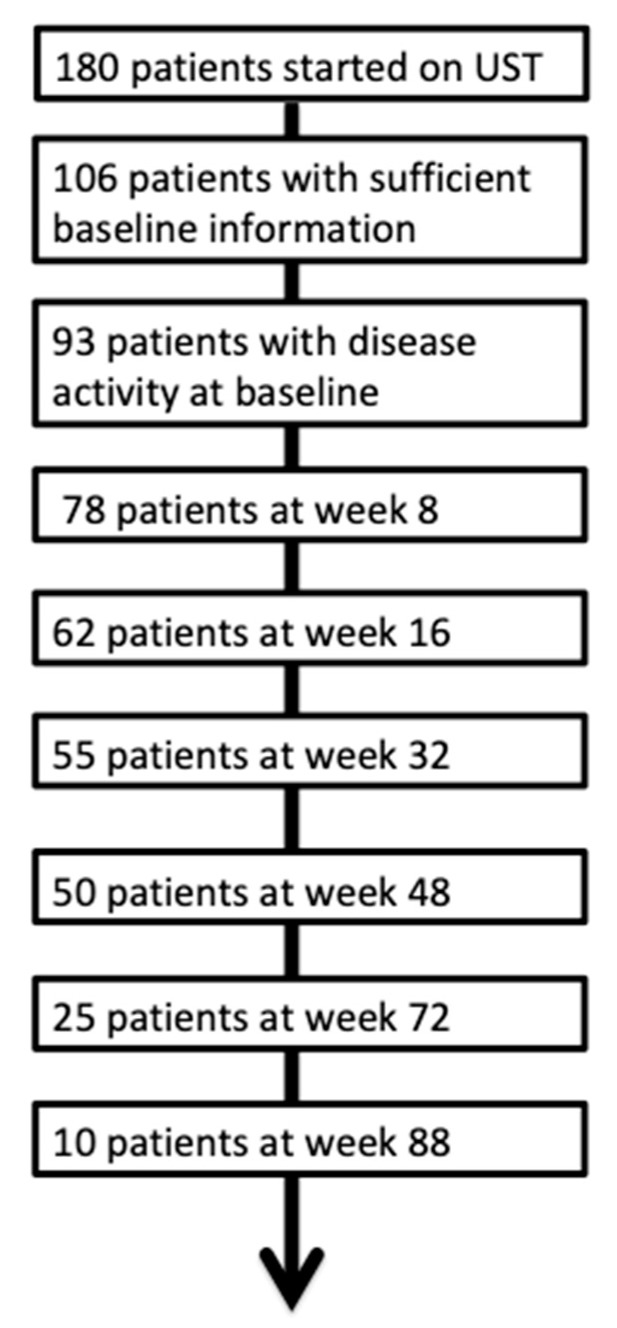
Flow chart of the overall population.

A combined response was defined as an HBI reduction by three points, fCal reduction of 50% or a value below 250 µg/g. For an overview of the number, patients included, please see Figure 1. 

At week 8, disease activity data was available for 78 patients. Twenty-three patients (24.7%) were in remission at week 8. Fifty-one patients (54.8%) showed a response at week 8. Sixty-two patients were included in the efficacy evaluation of week 16. Thirty-seven patients (39.8%) showed a response to UST and 19 patients (20.4%) a remission. Of the 27 patients that did not respond to UST at week 8, nine (33.3%) showed a late response to UST at week 16.

**Figure 2:**
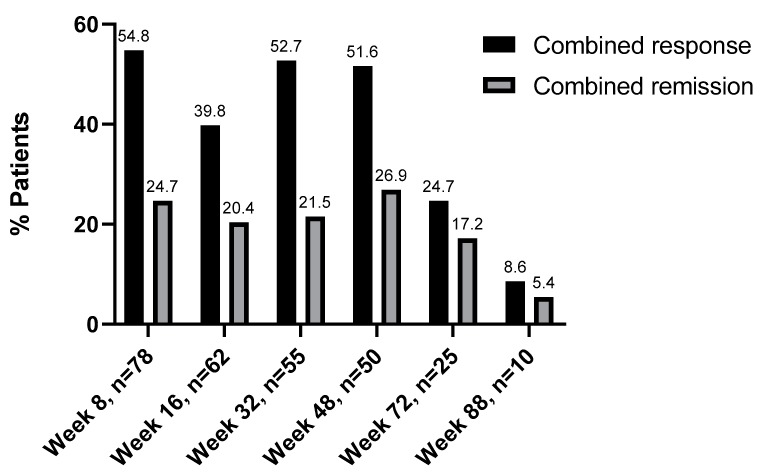
Combined effectiveness of ustekinumab. Response/Remission rates are shown in percentages (%).

At week 32, data were available for 55 patients. A total of 49 patients (52.7%) showed a response to UST, and 20 patients (21.5%) were in remission. At the week 48 evaluation 50 patients could be included. Here, 48 patients (51.6%) showed a response to UST and 25 (26.9%) were in remission. Information concerning UST efficacy was available for 25 patients at week 72. At this time point 24.7% (*n* = 23) patients responded to UST, with 17.2% (*n* = 16) being in remission. Last, we had ten documented cases for the 88-week time points. Here, eight patients (8.6%) responded to UST, and 5 (5.4%) were in remission. Of the 24 patients that were in remission at week 8, nine patients (75%) were still in remission at week 48 (Figure 2).

### 3.4. Comparing the Evaluation of Clinical and Combined Disease Activity

**Figure 3:**
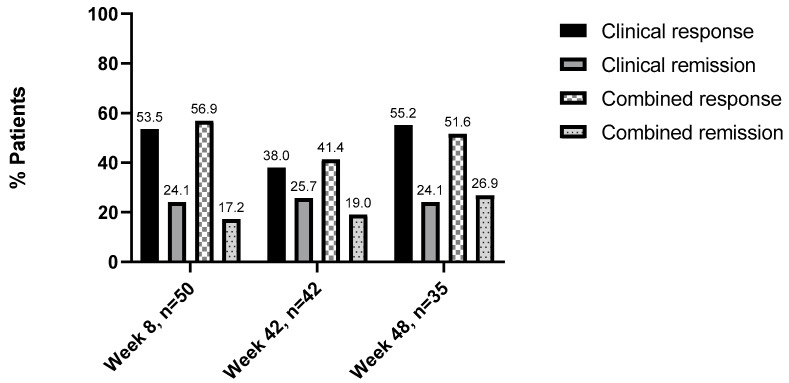
Comparison of clinical versus combined effectiveness of ustekinumab: Response/remission rates are shown in percentages (%).

Data on clinical activity was available for 75 patients out of the initial 106 patients at baseline. Of those, 58 patients had a disease activity and were included in the subanalysis of clinical versus combined response and remission. At week 8, data for clinical and combined disease activity was available for 50 patients. Thirty-one (53.5%) patients showed a clinical response to UST and 14 (24.1%) were in remission. When considering combined disease activity, 35 (56.9%) patients responded to UST, and 10 (17.2%) were in remission. At week 16, data were available for 42 patients. Here, 22 (38%) patients showed clinical response UST and 15 (25.7%) were in remission. For combined disease activity, 24 (41.4%) patients responded to UST and 11 (19%) were in remission. Last, at week 48, 35 patients were included in this subanalysis. Here, 32 (55.2%) patients clinically responded to UST, and 14 (24.1%) were in remission. When evaluating combined disease activity, 36 (62.1%) patients responded to UST and 15 (25.9%) were in remission (Figure 3).

### 3.5. Steroid-Free Remission and Response at Week 8 and 48

**Figure 4:**
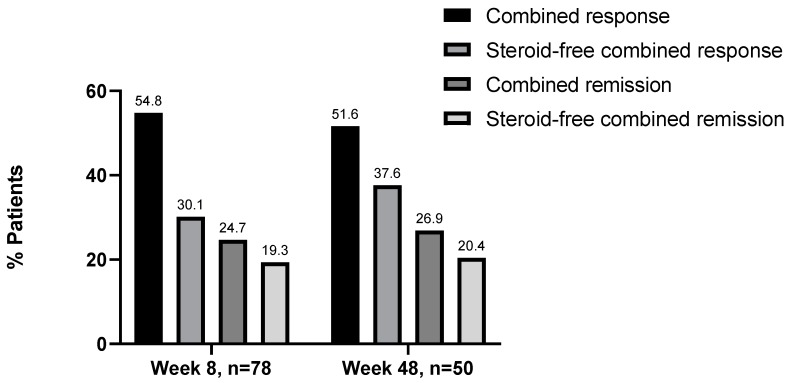
Combined effectiveness of ustekinumab: Steroid-free response/remission rates are shown in percentages (%).

Thirty-seven patients with disease activity at baseline were documented. At week 8, 31 patients still received concomitant steroid treatment. Of these patients, 23 patients (74.2%) showed a response to UST and 6 (19.4%) were in remission. The remaining patients at week 8 (*n* = 49) did not have concomitant steroid treatment. Here, 28 patients (30.1%) showed a response to UST and 18 (19.3%) were in remission. At week 48, 12 patients received concomitant steroid treatment. Of these patients, eleven patients showed a response to UST and five were in remission. The remaining patients at week 48, 38 patients did not have concomitant steroid treatment. Here, 35 patients (37.6%) showed a response to UST and 19 (20.4%) were in remission (Figure 4).

**Figure 5:**
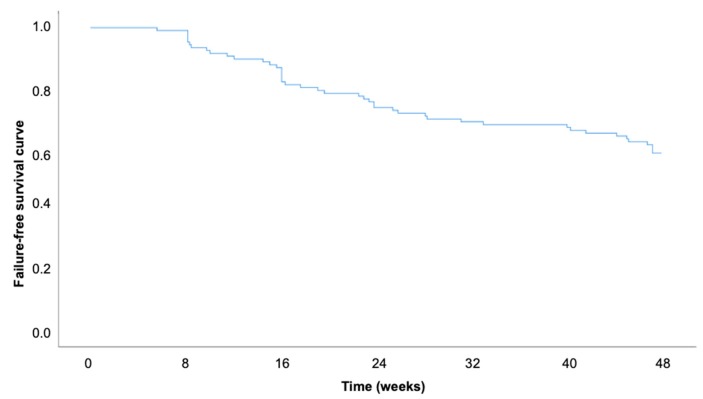
Kaplan-Meier curve for the probability of maintained ustekinumab treatment up to week 48.

The probabilities for maintained UST treatment were 93.4% (SE ± 2.4%) (for week 8, 81% SE ± 3.8%) for week 16, and 58.5% (SE ± 4.8%) for week 48 (Figure 5): For this calculation, all patients with sufficient baseline information (*n* = 106) were included.

### 3.6. Adverse Events, Non-Responders, and Re-Inductions

Eight patients stopped UST due to a secondary loss of response. Four of these patients received a re-induction with UST intravenously with the same dose as the initial one. Six patients underwent surgery, entailing two colectomies and one partial small intestine resection. The vast majority of patients received UST in an 8-week interval; thus, the respective time points for efficacy evaluation were chosen. UST was generally well tolerated. Adverse events were reported in three cases, with none leading to a UST treatment discontinuation. Two patients had an abscess formation in need of drainage, and one patient reported palpitations and a reduced sex drive. 

### 3.7. Predictors for Clinical Remission at Week 48 Follow-Up

Univariate and multivariate regression analyses were performed to determine parameters possibly associated with clinical remission and response at follow-up. The analyses were conducted for all patients with disease activity at baseline. Parameters evaluated amongst others included CRP, upper gastrointestinal manifestation, anti-TNFα exposure, and steroids at baseline. For simplification, not all evaluated parameters are displayed in Table 2. We included only significant parameters in the univariate analysis and a selection of nonsignificant parameters. A remission at week 8, a response to UST at week 16, male gender, and penetrating disease behavior were associated with remission in week 48 in the univariate analysis. For remission at week 8 and response at week 16, a positive association was observed.

## 4. Discussion

In our study, we provide real-world data on short- and long-term efficacy of UST in the treatment of patients with CD. Most patients in our cohort were anti-TNFα experienced (96.2%), and 34.4% had previously received both anti-TNFα and anti-Integrin medication. Response and remission rates at week 8 were reported for 54.8% and 24.7% of the patients, respectively. After 48 weeks of treatment, 51.6% of the patients showed a response to UST and 26.9% were in remission. For some patients, follow-up data were available up to 88 weeks. Only few adverse events were reported in this study, with none leading to end of treatment, indicating that long-term treatment with UST is safe and feasible.

When comparing our results with the results of the UNITI trials [16,17] and other real-world cohorts [7,10,11,12], differences in study design and efficacy results can be observed. 

In the UNITI-1 trial, short-term (Week 8) and long-term (Week 52) remission rates were assessed and were 20.8% and 41.1%, respectively. In comparison, in our study, rates for short-term (Week 8) and long-term (Week 48) remission were 24.7% and 26.9%. The observed difference in long-term remission rates could be because we assessed effectiveness with the help of both clinical and biochemical parameters for disease activity. Furthermore, our study cohort was not pre-selected (i.e., high dose steroids therapy, recent TNF-treatment, suspected need for surgery in the near future, draining stoma, or ostomy) as it is crucial for clinical trial [17]. Furthermore, it has to be noted that the majority of our patients has been previously exposed to TNF antagonists, whereas in IM-UNITI, approximately 40% of patients had no history of TNF antagonists [18]. The fact that UST was mainly a last-line therapy in our study could have led to maintaining some patients on UST without significant signs for response or remission, as no other option was available.

Two recent real-world studies from Belgium [10] and the Netherlands [12] report clinical response and remission rates in part comparable to our data. Both studies focused on rates of clinical response and remission. These were assessed with the help of the HBI only. Biological remission and response were evaluated separately. Liefferinckx et al. [10] reported short-term clinical response and remission rates of 59.2% and 28.2% and long-term response and remission rates of 42.1% and 25.7%, respectively. Biemans et al. [12] reported short-term clinical response and remission rates of 47.7% and 30.7% and long-term response and remission rates of 42.2% and 39.4%. When comparing both studies, note that the study of Biemans et al. [12] was conducted prospectively and that they calculated percentages from the number of patients available at the respective time point, whereas Liefferinckx et al. [10] calculated percentages from the baseline cohort. Although the dropout rate in Biemans’s study was low, this could result in slightly higher response and remission rates [12].

Both authors also assessed rates of biological response with the help of biochemical parameters. Liefferinckx et al. [10] reported a biological response rate of 41.1% at week 8, determined with the help of CRP, whereas Biemans et al. [12] reported remission biochemical short- and long-term remission rates of 23.9% and 26.4%. Therefore, when comparing the data for clinical and biological remission in these studies, it can be observed that rates for clinical remission are higher. However, note that biological remission was defined differently in both studies, only Biemans et al. took fCal in addition to CRP into consideration [12].

In our study, the response and remission rates were slightly lower, as we assessed UST efficacy with the help of both clinical activity indices (HBI) and biochemical parameters (CRP and fCal). When comparing clinical and combined response and remission at weeks 8, 16, and 48, we observed that when considering only clinical activity indices, the number of patients in remission was slightly overestimated. Interestingly, for the response we observed that evaluating both clinical and biochemical parameters, aided in a more differentiated assessment of the patient’s status. We, therefore, decided on combining the evaluation for clinical and biochemical response and remission as we observed that when both parameters were available, some cases showed relevant discrepancies between clinical and biochemical parameters (i.e., high fCal and/or CRP and normal HBI or vice versa). In these cases, the patient was not considered to be in remission. 

Combined effectiveness assessment is a novel approach, as most studies either only report clinical efficacy or differentiate between the biological/biochemical and clinical remission. However, we believe that both should be taken into consideration ideally in concert with endoscopic evaluation to paint a more reliable picture.

In the univariate analysis remission at week 8, response occurred at week 16. Male gender and penetrating disease behavior were associated with remission at week 48. Multivariate analysis was not conducted due to the sample size.

Few significant adverse events or side-effects were reported, with none of the leading to treatment discontinuation. This is in line with data from the PSOLAR registry [19] as well as IM-UNITI LTE [18] and several real-world studies [10,11].

Limitations of our study are mainly the retrospective multicenter design. Therefore, not all clinical and biochemical parameters were assessed for each patient, and especially the clinical assessment could be influenced by the treating clinician’s evaluation. Furthermore, the high loss to follow-up rate in our study is a major limitation. The participants were not required to visit their doctor for each time point. Some patients thus received prescriptions for UST and self-administered UST; therefore, we did not obtain data in these cases at specific relevant time points. Another explanation might be that some patients were included towards the end of the observational period and were thus not observed for their entire treatment period (i.e., no data for week 48 was available as these patients were just recently started on the medication). These aspects could partly explain the reduction in patients from baseline to week 48 and could overestimate the discontinuation/lost to follow-up rate in our study. When calculating the Kaplan–Meier curve, all patients with adequate baseline information (*n* = 108) were included. Patients that were observed for less than 16 weeks were considered as censored cases.

Furthermore, data on endoscopic response is missing in our study, which could aid in a more objective evaluation of remission and response. Last, as we evaluated response and remission with a combination of clinical and biochemical parameters, our study is less comparable to other real-world studies on this subject.

Our study has its merits, as it provides further insight on UST efficacy assessed with a combination of clinical and biochemical parameters. To conclude, we show the effectiveness and safety of UST in a large, therapy-experienced, and unselected real-world cohort.

## 5. Conclusions

We provided data on UST efficacy in multi-treatment experienced patients. UST was well tolerated. A combination of clinical and biochemical activity indices help to improve response and remission assessment. Response at week 16 was predictive for remission at week 48.

## Figures and Tables

**Table 1:** Baseline characteristics of included patients.

	***n* = 106**
Female gender (%)	62; (58.5)
Age (years), median (range)	39.5; (19–73)
Disease duration (years), median (range)	11; (2–39)
Median follow-up (weeks), median (95% CI)	49.1 (95% CI 42.03–56.25)
Vienna Classification	
Age at diagnosis, *n*	
A1 (<40 year), *n* (%)	75 (79.8)
A2 (≥40 year), *n* (%)	19 (20.2)
Disease location	
L1 (ileal), *n* (%)	21 (22.8)
L2 (colonic), *n* (%)	16 (17.4)
L3 (ileocolonic), *n* (%)	35 (38)
L4 (upper GI), *n* (%)	20 (21.8)
Phenotype	
B1 (inflammatory), *n* (%)	33 (34.4)
B2 (stenosing), *n* (%)	20 (20.8)
B3 (penetrating), *n* (%)	43 (44.8)
Previous anti-TNFα therapy, *n* (%)	102 (96.2)
≥1 TNFα, *n* (%)	55 (51.9)
≥2 TNFα, *n* (%)	46 (43.4)
None, *n* (%)	5 (4.7)
Previous anti-Integrin therapy, *n* (%)	36 (34.4)
Exposure to both *, *n* (%)	36 (34.4)
Previous immunosuppressants, *n* (%)	95 (89.6)
Steroids at baseline, *n* (%)	38 (35.8)
Baseline disease activity, *n* (%)	
Remission	13 (12.3)
Mild	11 (10.4)
Moderate	45 (42.5)
Severe	37 (34.9)
HBI score, median (range)	8; (0–26)
CRP, median (range)	0.95; (0.1–12.2)
fCal µg/g, median (range)	539; (5–2100)

Harvey–Bradshaw Index (HBI), C-reactive Protein (CRP), Fecal Calprotectin (fCal), * both = anti-TNF and anti-Integrin therapy.

**Table 2:** Logistic regression analysis for factors associated with remission at week 48.

	Univariate Analysis	
	*p*-Value	OR (95% CI)
Age	0.854	1.04 (0.96–1.05)
Male Gender	0.031	0.26 (0.08–0.88)
Remission week 8	0.025	4.75 (1.21–18.58)
Response week 16	0.003	10.52 (2.27–48.75)
Steroids at baseline	0.66	0.78 (0.25–2.40)
Penetrating behavior (B3)	0.03	0.25 (0.07–0.89)
Anti-Integrin therapy	0.61	0.73 (0.22–2.43)
HBI > 4	0.13	0.25 (0.43–1.45)
fCal > 250 µg/g	0.48	1.90 (0.31–11.61)

Odds ratio (OR), confidence interval (CI).
